# PIN1 Protects Hair Cells and Auditory HEI-OC1 Cells against Senescence by Inhibiting the PI3K/Akt/mTOR Pathway

**DOI:** 10.1155/2021/9980444

**Published:** 2021-06-02

**Authors:** Yanzhuo Zhang, Zhe Lv, Yudong Liu, Huan Cao, Jianwang Yang, Baoshan Wang

**Affiliations:** ^1^Department of Otorhinolaryngology, The Second Hospital of Hebei Medical University, No. 215 West Heping Road, Shijiazhuang 050000, China; ^2^Department of Otorhinolaryngology, Hebei General Hospital, Shijiazhuang 050051, China

## Abstract

A growing amount of evidence has confirmed the crucial role of the prolyl isomerase PIN1 in aging and age-related diseases. However, the mechanism of PIN1 in age-related hearing loss (ARHL) remains unclear. Pathologically, ARHL is primarily due to the loss and dysfunction of hair cells (HCs) and spiral ganglion cells (SGCs) in the cochlea. Therefore, in this study, we aimed to investigate the role of PIN1 in protecting hair cells and auditory HEI-OC1 cells from senescence. Enzyme-linked immunosorbent assays, immunohistochemistry, and immunofluorescence were used to detect the PIN1 protein level in the serum of ARHL patients and C57BL/6 mice in different groups, and in the SGCs and HCs of young and aged C57BL/6 mice. In addition, a model of HEI-OC1 cell senescence induced by H_2_O_2_ was used. Adult C57BL/6 mice were treated with juglone, or juglone and NAC, for 4 weeks. Interestingly, we found that the PIN1 protein expression decreased in the serum of patients with ARHL, in senescent HEI-OC1 cells, and in the cochlea of aged mice. Moreover, under H_2_O_2_ and juglone treatment, a large amount of ROS was produced, and phosphorylation of p53 was induced. Importantly, PIN1 expression was significantly increased by treatment with the p53 inhibitor pifithrin-*α*. Overexpression of PIN1 reversed the increased level of p-p53 and rescued HEI-OC1 cells from senescence. Furthermore, PIN1 mediated cellular senescence by the PI3K/Akt/mTOR signaling pathway. In vivo data from C57BL/6 mice showed that treatment with juglone led to hearing loss. Taken together, these findings demonstrated that PIN1 may act as a vital modulator in hair cell and HEI-OC1 cell senescence.

## 1. Introduction

Age-related hearing loss (ARHL) or presbycusis is a prevalent disease in aging people [1, 2]. Hearing loss occurs in most people as they age. Approximately one-third of people over 65 years old are affected by hearing loss according to the World Health Organization. With the aging of the population worldwide, more than 500 million people suffer ARHL [3]. This condition significantly affects the daily communication of older people, and has been shown to be associated with predisposing cognitive impairment and dementia. ARHL is characterized by an age-dependent decline in auditory function. Pathologically, this condition is primarily due to the loss of hair cells and spiral ganglion cells (SGCs) in the cochlea [4]. However, the exact mechanisms of ARHL remain largely unknown.

Peptidyl-prolyl *cis*/*trans* isomerase (PIN1) is a novel postphosphorylation signaling regulator. PIN1 regulates proteins by controlling the structure of phosphoproteins by mediating the isomerization of specific phosphorylated Ser/Thr-Pro motifs. As a molecular timer, PIN1 not only controls cell cycle progression and cell division but also regulates cellular senescence [5–8]. Loss of PIN1 in cultured cells induced senescence [8]. PIN1 knock-out mice showed a senescent phenotype [9]. PIN1 expression declined in the myocardium with aging [10]. All these results indicate that PIN1 is an important antiaging molecule. However, the role of this critical regulatory molecule in hair cells has not been previously examined.

Many previous studies have shown that the PI3K/Akt/mTOR pathway is an important pathway in regulating autophagy and apoptosis in the inner ear cochlea [11–14]. Liu et al. [11] demonstrated that the antioxidant enzyme PRDX1 triggers autophagy in spiral ganglion neurons through activation of the PTEN-Akt signaling pathway. Rapamycin alleviated cisplatin-induced ototoxicity by promoting autophagy [11, 15]. In Fus1 KO mice, researchers found dysregulation of Akt and activation of mTOR in cochleae [16]. Although the PI3K/Akt/mTOR pathways are associated with sensory hair cell survival, the mechanisms through which PI3K/Akt/mTOR regulate hair cell senescence are not fully defined.

HEI-OC1 cells are a hair-cell-like cell line that maintain hair cell characteristics and have been extensively used in many previous studies to investigate the protective mechanism of hair cells [17–21]. Therefore, in this study, we used aging C57BL/6 mice and H_2_O_2_-treated HEI-OC1 cells to determine the role and mechanism of PIN1 in the senescence of hair cells and HEI-OC1 cells.

## 2. Materials and Methods

### 2.1. Participants

The participants were selected from the Second Hospital of Hebei Medical University. Participants aged 30 to 80 years old without hearing loss were included in the control group (27 people aged 30-60 years old; 25 people aged 65-80 years old). In the study, 20 patients who were clinically determined to have age-related SNHL were included as the study group. The exclusion criteria were severe diseases, such as cancer, dementia, and psychiatric disorders.

### 2.2. Pure Tone Audiometry

Procedures were conducted in a soundproof booth. Sounds were delivered via earphone (TDH-50P). Six frequencies (250 Hz to 8000 Hz) were tested in routine pure tone audiometric examination (OB922 Audiometer, Madsen, Ltd., Denmark). The mean threshold of each frequency was calculated in both ears for each subject individually. The mean individual subject thresholds of 250, 500, and 1000 Hz were averaged to obtain the average pure tone hearing level of low frequencies (PTA) and that of 2000, 4000, and 8000 Hz as the average pure tone hearing level of high frequencies (PTA). HL was defined as PTA > 30 dB.

### 2.3. Animals

C57BL/6 mice were purchased from the Laboratory Animal Center, Charles River (Beijing China). Mice were divided into two groups: the young group (2 months old) and the old group (12 months old). Two-month-old mice were randomly divided into the control group, the juglone group, the juglone + NAC group, and the DMSO group. There were 6 mice per group. All experiments were performed according to protocols approved by the Animal Research Center, Hebei Medical University.

### 2.4. Enzyme-Linked Immunosorbent Assay (ELISA)

After centrifugation, serum samples were immediately frozen at -80°C for further analysis. Human PIN1 ELISA kits (Cat# ZC-32747), human ROS ELISA kits (Cat# ZC-33336), mouse PIN1 ELISA kits (Cat# ZC-54798), and mouse ROS ELISA kits (Cat# ZC-38260) were from Shanghai ZCIBIO. Then, the concentrations were measured according to the manufacturer's instructions. The absorbance at 450 nm was measured using a Synergy2 Automated Enzyme-Linked Immunosorbent Assay (Thermo Fisher Scientific, Inc., USA).

### 2.5. Cell Culture and Cell Transfection

HEI-OC1 cells (the House Ear Institute-Organ of Corti 1 cell line) were grown under permissive conditions (33°C, 10% CO_2_) in high-glucose Dulbecco's modified Eagle's medium (DMEM; Gibco USA) containing 10% fetal bovine serum (FBS; Gibco BRL) without antibiotics.

HEI-OC1 cells were cultured in 6-well plates and transfected when the cell fusion degree was 70-90%. The Lipofectamine 3000 Reagent was diluted with serum-free DMEM. A master mix of DNA was prepared by diluting DNA with serum-free DMEM medium, then adding the P3000 Reagent, and mixing. The diluted DNA and Lipofectamine 3000 Reagent were mixed (1 : 1) and incubated for 15 minutes at room temperature. DNA-lipid complexes were added to the cell supernatant and incubated for 2 days at 33°C.

### 2.6. Auditory Brainstem Response

All mice were anesthetized with an intraperitoneal injection (a mixture of ketamine-xylazine: 100 mg/kg ketamine and 10 mg/kg xylazine). Then, platinum needle electrodes were inserted at the vertex (reference electrode), behind the right ear (active electrode), and at the back (ground electrode) of the mice. Auditory brainstem responses (ABR) were measured in response to tone pips of 8, 12, 16, 20, 24, 28, and 32 kHz. ABR recordings were performed with a Tucker Davis Technologies (TDT) System III workstation running in a BioSigRP Soundbooth (IAC Acoustics). The hearing threshold was defined as the lowest intensity to generate a reproducible ABR waveform.

### 2.7. Immunohistochemistry

C57BL/6 mice were decapitated after the ABR tests. The temporal bones were dissected, and the cochleae were obtained and fixed with 4% paraformaldehyde at 4°C overnight and decalcified in 4% sodium ethylenediaminetetraacetic acid for 3 days at 4°C.

The cochleae were then dehydrated, processed, and embedded in paraffin. Paraffin-embedded specimens were cut to a thickness of 4 *μ*m. We selected morphologically intact slices to conduct immunohistochemistry staining. The selected 4 *μ*m sections were deparaffinized and rehydrated. Antigen recovery was performed by a microwave. Next, the sections were incubated with a primary antibody against PIN1 (10495-1-AP, Proteintech, USA) overnight at 4°C. The following day, after sequential incubation with a biotinylated secondary antibody and horseradish peroxidase conjugated streptavidin for 30 min at 37°C, the sections were stained with 3,3-diaminobenzidine (DAB). The slides were finally dehydrated, cleared, and mounted with coverslips. The negative controls were prepared by replacing the primary antibody with phosphate-buffered saline.

### 2.8. Immunofluorescence

After decalcification with 4% sodium EDTA solution for 3 days at 4°C, the cochleae were microdissected into three turns (apex, middle, and base). HEI-OC1 cells were fixed with 4% paraformaldehyde. The tissue and cell specimens were first permeabilized in Triton X-100 solution and then blocked with 10% normal goat serum for 30 min at room temperature. After incubation with primary anti-PIN1 (10495-1-AP, Proteintech, USA) antibody overnight at 4°C, the samples were then washed three times with PBS and incubated with secondary fluorescent antibody for 1 h at 37°C in the dark to detect the primary antibody, followed by incubation with Alexa Fluor 488-phalloidin for 1 h at room temperature in the dark.

### 2.9. Reagent Treatment

HEI-OC1 cells were seeded in six-well plates for each experiment for 24 h. Before H_2_O_2_ stimulation, the cells were pretreated with the PI3K inhibitor LY294002 (25 *μ*M, HY-10108/CS-0150, MCE, USA), the Akt activator SC79 (4 *μ*g/mL, B5663, APExBIO, USA) and the ROS scavenger NAC (2 mM, G1902071, Aladdin, China) for 1 h; the p53 inhibitor pifithrin-*α* (10 *μ*M, A4206, APExBIO, USA) was used for 24 h. The PIN1 inhibitor juglone (1, 5, and 10 *μ*M, STBH9858, Sigma-Aldrich, USA) was used for 45 min.

### 2.10. Senescence-Associated *β*-Galactosidase Stain

Cellular senescence-associated *β*-galactosidase (SA-*β*-Gal) staining was conducted using a senescence *β*-galactosidase staining kit (Beyotime Institute of Biotechnology, Shanghai, China) following the manufacturer's instructions. Before staining, cells were gently washed with PBS and fixed with fixing solution. After washing three times with PBS, 1 mL of staining solution was added to each well, sealed with parafilm, and incubated at 37°C without CO_2_ overnight.

### 2.11. Protein Extraction and Western Blotting

The cells were collected and lysed on ice with RIPA buffer and PMSF for 30 min. Detection of phosphorylated proteins requires the addition of phosphorylase inhibitors (04906845001, Roche Switzerland). The supernatant was centrifuged at 12,000 rpm for 20 min at 4°C. The BCA Protein Assay (PC0020, Solarbio, China) was used to determine the protein concentration. The protein extracts from the cells were separated by sodium dodecyl sulfate polyacrylamide gel electrophoresis and then transferred to polyvinylidene fluoride membranes. The membranes were blocked with 5% nonfat milk for 2 h at 37°C and then incubated overnight at 4°C with anti-PIN1 (dilution, 1 : 1000; 10495-1-AP, Proteintech, USA), p53 (dilution, 1 : 1000; 21891-1-AP, Proteintech, USA), p-p53 (dilution, 1 : 1000; 9284, Cell Signaling Technology, USA), p21 (1 : 1000, dilution, 1 : 1000; 27296-1-AP, Proteintech, USA), p-16 (1 : 1000, 10883-1-AP, Proteintech, USA), Akt (1 : 1000, ab8805, Abcam, USA), p-Akt (1 : 1000, ab81283, Abcam, USA), mTOR (1 : 1000, 2983, Cell Signaling Technology, USA), p-mTOR (1 : 1000, 5536, Cell Signaling Technology, USA), and GAPDH (1 : 2000 Proteintech, USA) antibodies. ImageJ software was used to quantify the band intensity. The intensity value of each target band was normalized to that of GAPDH.

### 2.12. Statistical Analysis

All experiments were independently repeated at least three times. Data are presented as the mean ± SD and were analyzed with SPSS. Student's *t*-test and one-way ANOVA were used for statistical analysis. Values with *P* < 0.05 were considered significant.

## 3. Results

### 3.1. PIN1 and ROS Concentrations in Human and C57BL/6 Mouse Serum

**Figure 1:**
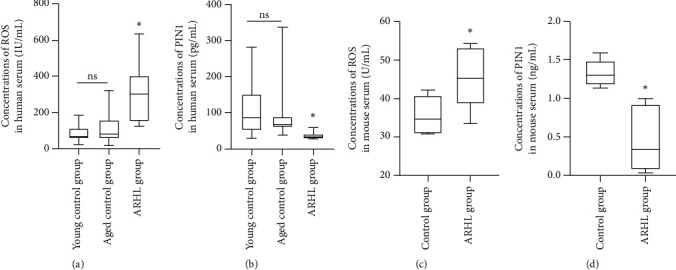
PIN1 and ROS concentrations in human and mouse sera. (a, c) ROS expression levels were increased in the patients of the ARHL group and old C57BL/6 mice (^∗^*P* < 0.05). (b, d) PIN1 expression levels were decreased in the patients and old C57BL/6 mice with ARHL (^∗^*P* < 0.05).

The serum concentrations of PIN1 and ROS were measured in all participants and C57BL/6 mice. ELISAs revealed that ROS expression levels were increased in the patients in the ARHL group and the old C57BL/6 mice (Figures 1(a) and 1(c)). PIN1 expression levels were decreased in the patients and in the old C57BL/6 mice with ARHL (Figures 1(b) and 1(d)).

### 3.2. Elevated Auditory Brainstem Response Threshold and Cell Senescence in the Cochleae of the Aged C57BL/6 Mice

**Figure 2:**
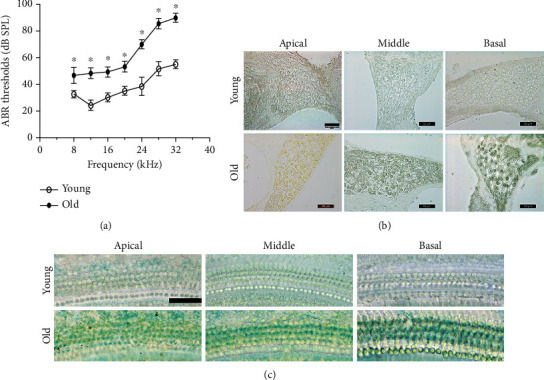
Hearing impairment in old mice at all frequencies and *β*-gal staining of the cochleae. (a) Compared with young mice, old mice showed significantly increased ABR thresholds at all frequencies (^∗^*P* < 0.05). (b) Representative senescence-associated *β*-galactosidase (SA-*β*-gal) staining of SGCs. (c) Representative senescence-associated *β*-galactosidase (SA-*β*-gal) staining of HCs. Scale bar = 50 *μ*m.

We examined the auditory function in young and old mice by measuring ABR. As shown in Figure 2(a), not only was the ABR threshold at a high frequency of the old mice significantly higher than that of the young mice but also that at middle and low frequencies was significantly higher in the old mice than the young mice, which indicated a decline in auditory function in the old mice. In addition, the results showed that senescence-associated *β*-galactosidase- (SA-*β*-gal-) positive cells in the aged mice were more abundant than those in the young mice in spiral ganglion cells (SGCs) (Figure 2(b)) and hair cells (HCs) (Figure 2(c)).

### 3.3. The Expression of PIN1 Is Downregulated in the Cochleae of the Aged C57BL/6 Mice

**Figure 3:**
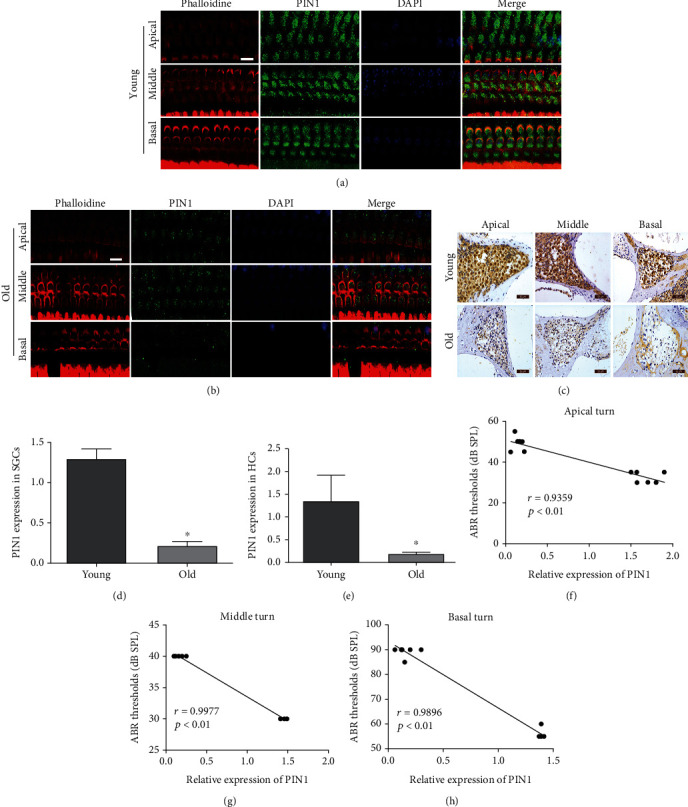
The expression of PIN1 is downregulated in the cochleae of aged C57BL/6 mice. (a, b, and e) Representative images of immunofluorescence staining of PIN1 in HCs from young and old animals (scale bar = 10 *μ*m). (c and d) Immunohistochemistry staining for PIN1 in the SGCs of young and old mice (scale bar = 50 *μ*m). (f–h) Linear analysis showed that the expression of PIN1 in the cochlea was negatively correlated with hearing threshold (^∗^*P* < 0.05).

We examined PIN1 expression in the cochlea of the young and old mice. IF and IHC analysis showed that the PIN1 protein was expressed in the cytoplasm and nucleus of HCs (Figures 3(a) and 3(b)) and SGCs (Figure 3(c)). PIN1 expression was markedly decreased in the old mice compared to the young mice (Figures 3(a)–3(e)). Linear analysis showed that the expression of PIN1 in the cochlea was negatively correlated with hearing threshold (Figures 3(f)–3(h)). Taken together, the results showed that PIN1 might be involved in ARHL.

### 3.4. Downregulation of PIN1 Expression in HEI-OC1 Cells Induced by H_2_O_2_

**Figure 4:**
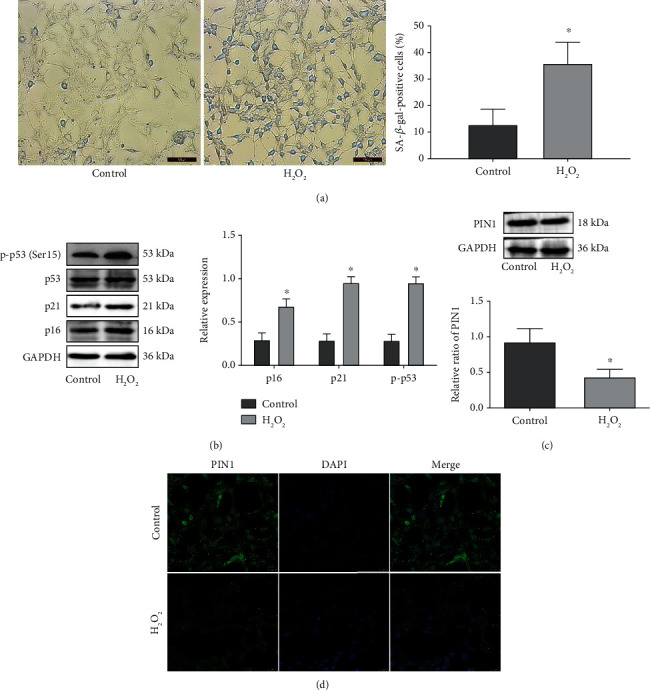
H_2_O_2_ induces premature senescence and decreases PIN1 expression in HEI-OC1 cells in vitro. (a) Representative senescence-associated *β*-galactosidase (SA-*β*-gal) staining of HEI-OC1 cells (scale bar = 100 *μ*m). (b) Western blots for the expression of p53, p-p53, p21, and p16. (c) Western blot for the expression of PIN1 in HEI-OC1 cells. (d) Immunofluorescence staining for PIN1 in HEI-OC1 cells (scale bar = 25 *μ*m) (^∗^*P* < 0.05).

The different concentrations of H_2_O_2_ on cell viability were detected by the MTS method. After 2 h of stimulation by H_2_O_2_, the relative cell survival rate was observed and IC50 = 1.17 mM was calculated (Supplementary Figure 1). Therefore, HEI-OC1 cells were exposed to H_2_O_2_ (1 mM for 2 h) to induce cellular senescence. Thereafter, senescence-associated SA-*β*-Gal staining was applied to detect senescent HEI-OC1 cells. As shown in Figure 4(a), the percentage of cells positive for SA-*β*-Gal was increased in the HEI-OC1 cells treated with H_2_O_2_. Additionally, H_2_O_2_ significantly elevated the expression of p-p53, p21, and p16 compared with that in the control group (Figure 4(b)). However, PIN1 protein expression, detected by Western blotting (Figure 4(c)) or immunofluorescence (Figure 4(d)), was significantly decreased in the HEI-OC1 cells treated with H_2_O_2_.

### 3.5. PIN1 Modulates HEI-OC1 Cell Senescence

**Figure 5:**
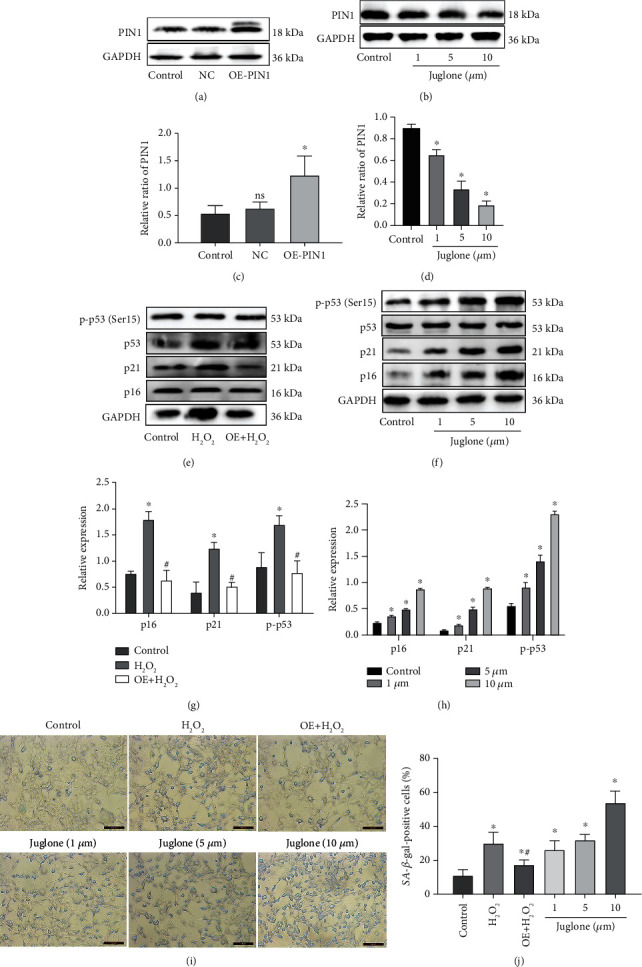
PIN1 modulates HEI-OC1 senescence. (a, c) HEI-OC1 cells were transfected with the HA-PIN1 plasmid. (b, d) HEI-OC1 cells were treated by juglone. (e–h) Western blots for the expression of p53, p-p53, p21, and p16 in HEI-OC1 cells. (i, j) SA-*β*-gal staining was used to detect the senescent state of HEI-OC1 cells (OE: overexpression-PIN1; ^∗^*P* < 0.05 vs. control group, ^#^*P* < 0.05 vs. H_2_O_2_ group. Scale bar = 100 *μ*m).

To better understand the role of PIN1 in the cellular senescence of HEI-OC1 cells, we transfected the HA-PIN1 overexpression plasmid into HEI-OC1 cells or treated HEI-OC1 cells with juglone, a PIN1 inhibitor [22]. The transfection effect is shown in Figures 5(a) and 5(c). As illustrated in Figures 5(e) and 5(g), PIN1 overexpression caused a significant reduction in the expression of p-p53, p21, and p16. The percentage of SA-*β*-gal-positive cells was also reduced in the PIN1 overexpression group (Figures 5(i) and 5(j)). In contrast, juglone inhibited PIN1 in a concentration-dependent manner (Figures 5(b) and 5(g)) and accelerated senescence in HEI-OC1 cells in a concentration-dependent manner (Figures 5(f), 5(h), 5(i), and 5(j)). However, these changes were not found in the different doses of solvent control group (Supplementary Figures 2(A) and 2(B)).

### 3.6. PIN1 Mediates HEI-OC1 Cell Senescence by Affecting the PI3K/Akt/mTOR Signaling Pathway

**Figure 6:**
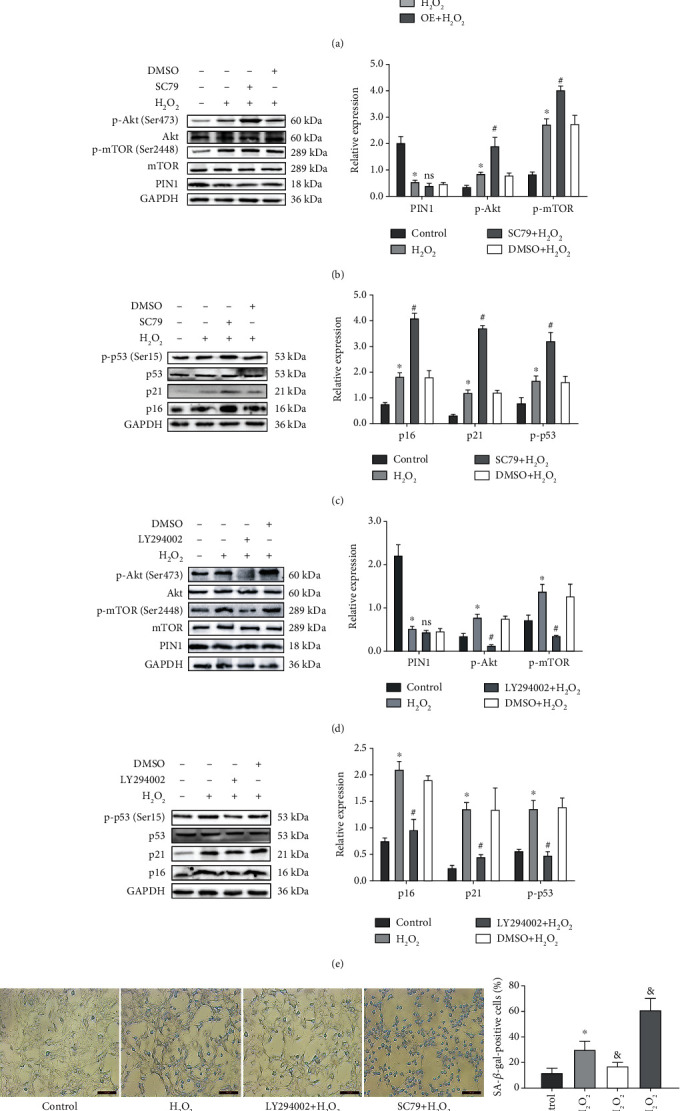
PIN1 mediates HEI-OC1 cell senescence by affecting the PI3K/Akt/mTOR signaling pathway. (a) Western blots for the expression of p-Akt, Akt, p-mTOR, and mTOR in HEI-OC1 cells after transfection with the HA-PIN1 plasmid. (b) Western blots for the expression of p-Akt, Akt, p-mTOR, mTOR, and PIN1 in HEI-OC1 cells after pretreatment with SC79 (Akt activator). (c) Western blots for the expression of p53, p-p53, p21, and p16 after pretreatment with SC79. (d) Western blots for the expression of p-Akt, Akt, p-mTOR, mTOR, and PIN1 in HEI-OC1 cells after pretreatment with LY294002 (an inhibitor of PI3K/Akt). (e) Western blots for the expression of p53, p-p53, p21, and p16 after pretreatment with LY294002. (f) SA-*β*-gal staining for HEI-OC1 cells (^∗^*P* < 0.05 vs. control group, ^#^*P* < 0.05 vs. DMSO+H_2_O_2_ group, ^&^*P* < 0.05 vs. H_2_O_2_ group. Scale bar = 100 *μ*m).

**Figure 7:**
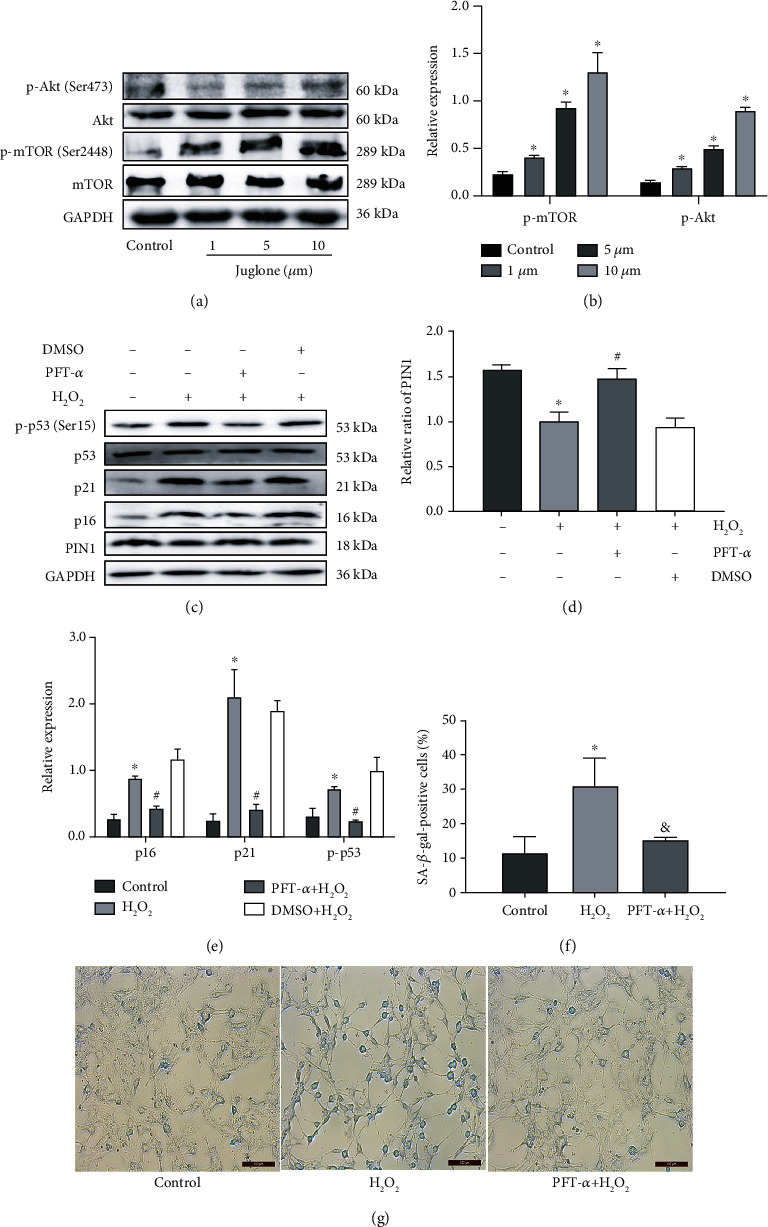
H_2_O_2_ affects the expression of PIN1 through p53, which in turn affects senescence. (a, b) Western blots for the expression of p-Akt, Akt, p-mTOR, and mTOR in HEI-OC1 cells after treatment with juglone. (c–e) Western blots for the expression of p53, p-p53, p21, p16, and PIN1 after pretreatment with the p53 inhibitor pifithrin-*α* (PFT-*α*). (f, g) SA-*β*-gal staining for HEI-OC1 cells (^∗^*P* < 0.05 vs. control group, ^#^*P* < 0.05 vs. DMSO+H_2_O_2_ group, ^&^*P* < 0.05 vs. H_2_O_2_ group. Scale bar = 100 *μ*m).

Western blotting results showed that PIN1 overexpression rescued the H_2_O_2_-induced upregulation of p-Akt and p-mTOR expression in HEI-OC1 cells (Figure 6(a)). However, PIN1 inhibition induced upregulation of p-Akt and p-mTOR expression (Figures 7(a) and 7(b)), and the expression of p-Akt and p-mTOR did not change in the different doses of the solvent control group (Supplementary Figures 2(C) and 2(D)). Then, to further study the role of the PI3K/Akt/mTOR signaling pathway in HEI-OC1 cell senescence, we pretreated HEI-OC1 cells with SC79 (Akt activator) or LY294002 (Akt inhibitor) before H_2_O_2_ stimulation. The results showed that neither SC79 nor LY294002 changed the PIN1 protein levels (Figures 6(b) and 6(d)). SC79 upregulated the expression of p-p53, p16, and p21 (Figure 6(c)) and increased the number of SA-*β*-gal-positive cells (Figure 6(f)). However, LY294002 had an opposite effect compared with SC79 (Figures 6(e) and 6(f)). These results indicated that the protective role of PIN1 could be mediated by inhibiting the PI3K/Akt/mTOR pathway in senescent HEI-OC1 cells.

### 3.7. H_2_O_2_ Affects the Expression of PIN1 through p53, which In Turn Affects Senescence

To further investigate the regulatory mechanism of PIN1 in ARHL, we used the p53 inhibitor pifithrin-*α* (PFT-*α*) to evaluate whether H_2_O_2_ affects the expression of PIN1 through p53. When HEI-OC1 cells were incubated with PFT-*α*, the p-p53 protein level was decreased, but the PIN1 protein level significantly increased (Figures 7(c) and 7(d)). In addition, we investigated the expression of senescence-associated proteins. The results showed that pretreatment of the cells with PFT-*α* caused a significant decrease in the expression of p21 and p16 (Figures 7(c) and 7(e)), and the percentage of SA-*β*-gal-positive cells was also reduced (Figures 7(f) and 7(g)). The results suggested that H_2_O_2_ inhibited the expression of PIN1 by upregulating p53 expression, which in turn affected senescence.

### 3.8. H_2_O_2_ Affects the Expression and Activity of p53 through ROS and Then Affects Senescence

**Figure 8:**
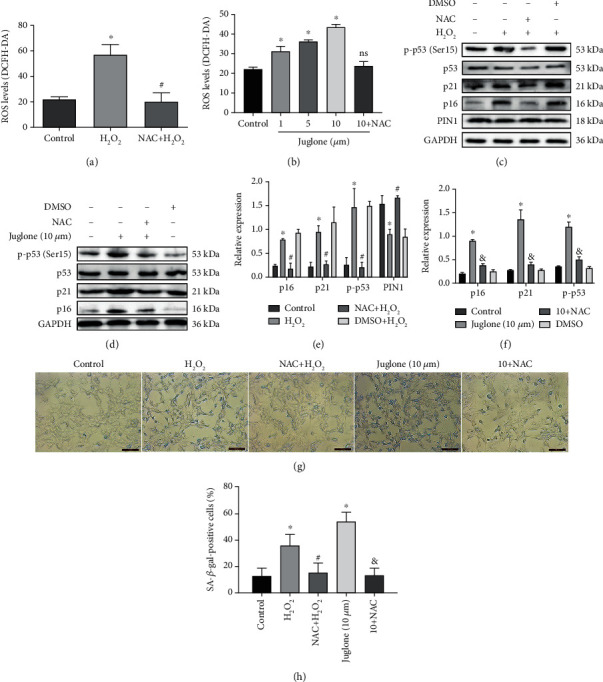
H_2_O_2_ affects the expression and activity of p53 through ROS and then affects senescence. (a, b) Intracellular ROS levels were measured by DCFH-DA in HEI-OC1 cells (ns: nonsignificant; ^∗^*P* < 0.05 vs. control group, ^#^*P* < 0.05 vs. H_2_O_2_ group). (c–f) Western blots for the expression of p53, p-p53, p21, p16, and PIN1 after pretreatment with the ROS inhibitor N-acetyl-L-cysteine (NAC) (^∗^*P* < 0.05 vs. the DMSO group, ^#^*P* < 0.05 vs. H_2_O_2_ group, ^&^*P* < 0.05 vs. the juglone group). (g, h) SA-*β*-gal staining for HEI-OC1 cells (^∗^*P* < 0.05 vs. the control group, ^#^*P* < 0.05 vs. the H_2_O_2_ group, ^&^*P* < 0.05 vs. the juglone group. Scale bars = 100 *μ*m).

In the model of senescence and in the juglone treatment group, high levels of ROS were produced (Figures 8(a) and 8(b)). To detect whether ROS affect the expression and activity of p53, we used the ROS inhibitor N-acetyl-L-cysteine (NAC). The results showed that the production of ROS almost recovered to the level of the control group after NAC treatment (Figures 8(a) and 8(b)). NAC led to a restoration of p-p53 levels (Figures 8(c)–8(f)). In addition, when cells were pretreated with NAC, the expression level of PIN1 was increased (Figures 8(c) and 8(e)), and the expression levels of p21 and p16 were decreased (Figures 8(c)–8(f)). The percentage of SA-*β*-gal-positive cells was also reduced by NAC treatment (Figures 8(g) and 8(h)).

### 3.9. Juglone Treatment Results in C57BL/6 Mouse Hearing Loss

**Figure 9:**
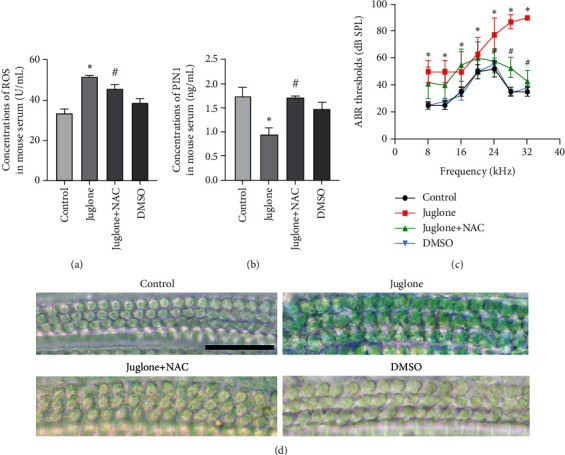
Juglone treatment induces C57BL/6 mouse hearing loss. (a) ROS level in the C57BL/6 mice serum. (b) PIN1 levels in the C57BL/6 mice serum. (c) Compared with the other groups, the juglone-treated group had significantly increased ABR thresholds at all frequencies, especially at high frequencies. Compared with the juglone-treated group, the juglone + NAC group had significantly decreased ABR thresholds at high frequencies. (d) In the basal turn hair cells, SA-*β*-gal-positive cells in the juglone-treated group were greater than those in the other groups (^∗^*P* < 0.05 vs. the DMSO group; ^#^*P* < 0.05 vs. the juglone group. Scale bars = 50 *μ*m).

To further study whether the reduction in PIN1 expression could induce hair cell senescence, we treated the mice with juglone, or juglone and NAC at the same time. Intraperitoneal injection of juglone (1 mg/kg, three times a week for 4 weeks) and/or NAC (1.5 g/kg/d, oral, for 4 weeks) was performed. As shown in Figures 9(a) and 9(b), in the juglone-treated group, the ROS level expression increased in the serum compared with the control group and the juglone + NAC group. In the juglone + NAC treatment group, the PIN1 level expression increased in the serum compared with the juglone group. Moreover, in the juglone-treated group, ABR thresholds at all frequencies were increased, especially at high frequency. Compared with the juglone-treated group, the juglone + NAC group had significantly decreased ABR thresholds at high frequencies (Figure 9(c)). In the basal turn hair cells, there were more SA-*β*-gal-positive cells in the juglone-treated group than in the other groups (Figure 9(d)).

## 4. Discussion

Sensorineural hearing loss is caused by many factors that impair hair cell and spiral ganglion neuron function [23–28], including ototoxic drugs, genetic factors, aging, noise exposure, and chronic cochlear infections [29–33]. A growing amount of evidence has demonstrated that PIN1 plays critical roles in aging-associated diseases [34–39]. PIN1-deficient mice displayed signs of premature senility, such as rapid telomere shortening and loss of motor coordination and behavioral defects and neuronal loss [7]. It has been reported that overexpression of PIN1 rescues cellular senescence of atherosclerotic VSMCs and downregulates the expression of p53 and p21 [40]. Toko et al. [10] reported that knockdown of PIN1 led to cellular senescence, but overexpression of PIN1 inhibited senescence of cardiac progenitor cells. However, the role of PIN1 in ARHL has not been previously reported. For this research, PIN1 protein expression was markedly reduced in the serum of patients with ARHL, SGCs, and HCs of old mice and senescent HEI-OC1 cells. The expression of PIN1 in the cochlea was negatively correlated with the hearing threshold. In vitro, the overexpression of PIN1 inhibited the senescence of HEI-OC1 cells. Inhibited PIN1 accelerated senescence in HEI-OC1 cells. In vivo, we found that treatment with juglone led to hearing loss in C57BL/6 mice, especially at high frequencies. All of these results support our hypothesis that PIN1 modulates HEI-OC1 and hair cell senescence.

Astle et al. reported that the activation of the PI3K/Akt signaling pathway induces cell senescence, and mTOR is a related mediator [41]. In this signaling pathway, mTOR is a downstream target of the PI3K/Akt signaling pathway, and Akt is a positive regulator of mTOR [42]. Studies have shown that topical application of the mTOR inhibitor rapamycin can reduce p16 protein expression and cell aging in skin tissue [43]. Hu et al. [44] demonstrated that Akt/mTOR activity was increased upon aging in zebrafish retinas, and increased Akt/mTOR activity is a major player in age-related retinal neuropathy in zebrafish. It was also demonstrated that gentamicin resulted in the activation of Akt in the organ of Corti and in spiral ganglion cells [45]. The mTOR inhibitor rapamycin protected sensory hair cells against gentamicin. Furthermore, rapamycin attenuates noise-induced hair cell loss by reducing oxidative stress [46]. Nevertheless, other studies suggest that PI3K/Akt signaling is a protective mechanism of the inner ear. It was reported that PI3K/Akt is an endogenous protective mechanism of the inner ear. The activation of PI3K/Akt protects mice from cochlear injury and hearing damage induced by gentamicin and noise [47, 48]. Inhibition of mTOR results in auditory hair cell damage and decreased spiral ganglion neuron outgrowth [49]. Inhibition of the PI3K/Akt pathway can induce senescence and directly increase the expression of p21 [50, 51]. In this article, we showed that the expression of p-Akt and p-mTOR in HEI-OC1 cells treated with H_2_O_2_ and juglone substantially increased. Inhibition of the PI3K/Akt/mTOR pathway significantly alleviated H_2_O_2_-induced senescence in HEI-OC1 cells. These results suggest that the activation of Akt aggravated senescence in HEI-OC1 cells.

In multiple cancer types, many studies have shown that the expression levels of PIN1 and levels of Akt phosphorylation are strongly correlated. However, experiments have shown that in mouse periodontal tissues, PIN1 siRNA enhanced mTOR and Akt activation, which was attenuated by PIN1 overexpression [52]. In the present study, PIN1 overexpression caused a reduction in the expression of p-Akt. The inhibition of PIN1 caused upregulation of p-Akt and p-mTOR expression. Furthermore, the activation of Akt aggravated H_2_O_2_-induced senescence in HEI-OC1 cells. All of these results suggested that PIN1 protects HEI-OC1 cells from senescence by inhibiting the PI3K/Akt/mTOR signaling pathway.

p53 plays a crucial role in the process of cellular senescence. p53 initiates various stress responses, including cell cycle arrest and aging [53]. The overexpression of p53 promotes aging. Our data showed that the expression of p-p53 was significantly higher in the H_2_O_2_-induced HEI-OC1 cells than in the control cells. Jeong et al. [54] reported that endoplasmic reticulum (ER) stress decreased PIN1 expression through p53 activation, and overexpression of p53 observably decreased PIN1 expression in HCT116 cells. However, the regulators of PIN1 expression during aging are still not fully understood, especially in ARHL. In our study, when the cells were treated with the p53 inhibitor pifithrin-*α*, PIN1 expression was significantly increased, so we hypothesized that H_2_O_2_ might regulate PIN1 expression through p53. p-p53 expression was significantly decreased by PIN1 overexpression. These findings suggest that PIN1 and p53 regulate each other. However, the exact mechanism remains to be determined.

The gradual accumulation of ROS is a key mediator during the aging process. In the present study, H_2_O_2_ and juglone treatment resulted in an increase in ROS activity, indicating that H_2_O_2_ induced senescence via the production of ROS, and inhibition of PIN1 caused increased ROS production also. The ROS scavenger NAC could reduce ROS production, reverse changes in aging-related biomarkers, protect cells from ROS-induced cellular senescence, and indirectly inhibit mTOR [55–57]. In this study, we also found that NAC reduced the ROS production and reversed changes in aging-related biomarkers. In PDAC cells, silencing PIN1 significantly increased intracellular ROS [58]. In this study, we speculate that significantly decreased PIN1 expression may increase ROS production.

**Figure 10:**
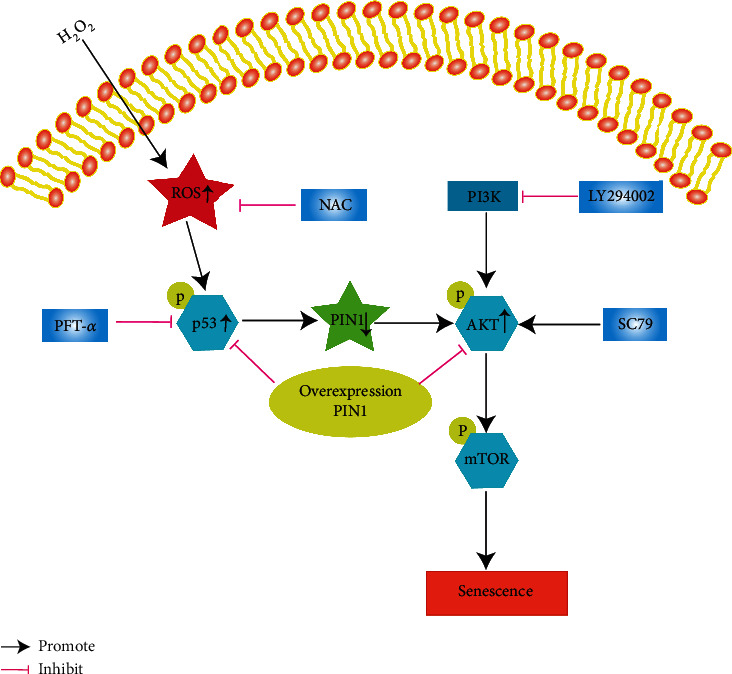
Schematic diagram of the observed effects and the antisenescence mechanism of PIN1 in the present study. H_2_O_2_ treatment resulted in oxidative damage, upregulated p-p53 expression, and activation of the PI3K/Akt/mTOR pathway. p-p53 negatively regulates PIN1 expression. Overexpression of PIN1 reversed the increase in p-p53 and inhibited the activation of the PI3K/Akt/mTOR pathway.

Collectively, under H_2_O_2_ treatment (or PIN1 inhibition), a large amount of ROS was produced, and the generation of ROS upregulated p-p53 expression. Then, p-p53 negatively regulated PIN1 expression, and overexpression of PIN1 reversed the increase in p-p53. More importantly, PIN1 mediated cellular senescence by affecting the PI3K/Akt/mTOR signaling pathway (Figure 10).

Above all, our findings, for the first time, demonstrated that PIN1 expression is downregulated in the serum of patients with ARHL, in the cochleae of aged C57BL/6 mice, and in senescent HEI-OC1 cells. The in vivo data showed that PIN1 inhibition led to hearing loss. However, only animal and cellular experiments were conducted, and clinical data and conclusions are lacking. These limitations need to be addressed in the future.

## Data Availability

Data can be found in the manuscript and supplementary information files.

## Supplementary Materials

Supplementary 1 Supplementary Figure 1: the different concentrations of H_2_O_2_ on cell viability were detected by the MTS method. After stimulation by H_2_O_2_ for 2 h, we observed the relative cell survival rate and calculated IC50 = 1.17 mM.

Supplementary 2 Supplementary Figure 2: (A, B) Western blot for the expression of p53, p-p53, p21, and p16 in HEI-OC1 cells after treatment with DMSO (0.1, 0.5, and 1 *μ*L); (C, D) Western blot for the expression of p-Akt, Akt, p-mTOR, and mTOR in HEI-OC1 cells after treatment with DMSO (0.1, 0.5, and 1 *μ*L).
